# Shear wave elastography of the supraspinatus tendon with early degeneration in asymptomatic type II diabetes mellitus patients: a multicenter study

**DOI:** 10.1186/s12891-025-08864-w

**Published:** 2025-07-04

**Authors:** Tong Wang, Yanni He, Wenhong Yi, Huiyu Xie, Dan Wang, Jie Zeng, Jinying Liang, Yufan Chen, Qiuyan Mo, Meijun Zhou, Sushu Li, Feifei Huang, Shaoming Liu, Liya Ma, Xueling Liu, Hongmei Liu

**Affiliations:** 1 Department of Ultrasound, Institute of Ultrasound in Musculoskeletal Sports Medicine, Guangdong Engineering Technology Research Center of Emergency Medicine, The Affiliated Guangdong Second Provincial General Hospital of Jinan University, Guangdong Guangzhou, 510317 People’s Republic of China; 2 The Second School of Clinical Medicine, Southern Medical University, Guangzhou, 510317 People’s Republic of China; 3 Department of Ultrasound, Chongzuo People’s Hospital, Guangxi Chongzuo, 532200 People’s Republic of China; 4 Department of Ultrasound, The First Affiliated Hospital of Guangxi University of Traditional Chinese Medicine, Guangxi Nanning, 530023 People’s Republic of China; 5 Department of Ultrasound, Affiliated Dongguan Hospital, Southern Medical University Dongguan People’s Hospital, Guangdong Dongguan, 523067 People’s Republic of China

**Keywords:** Supraspinatus tendon, Rotator cuff, Type II diabetes mellitus, Shear wave elastography, Tendinopathy

## Abstract

**Objectives:**

This study aimed to explore conventional ultrasound sonography and shear wave elastography (SWE) changes in the supraspinatus tendon in type 2 diabetes patients (T2DM) without shoulder pain. The factors influencing supraspinatus tendon thickness and shear wave velocity (SWV), which are measured by SWE, were also explored.

**Methods:**

This multicenter study recruited nonshoulder pain individuals, including healthy and diabetic patients from March 2021 to October 2022. Propensity score matching was used to overcome selection bias. The thickness and SWV of the bilateral supraspinatus tendons were measured, and their influencing factors were evaluated via regression analysis. The sonography and blood flow signals were observed and compared via χ2 tests.

**Results:**

A total of 107 T2DM patients and 270 healthy people were enrolled (mean age, 42 years ± 14, 162 men). Although 59.8% of T2DM patients without shoulder pain, they were likely to have degenerative ultrasonic image and a lower SWV (-0.529 [-0.827, -0.232] vs. 1; *p* = 0.001) than healthy people, but no difference was detected for uneven thickening of the tendon (*p* = 0.055) or blood flow signals (*p* = 0.060).

Furthermore, subjects > 50 years old, with a BMI ≥ 25.0 and who were of Chinese Zhuang population had thicker supraspinatus tendons than did the controls. Chinese Han population and regular upper limb exercise subjects had greater tendon SWV than did controls.

**Conclusions:**

SWE is a repeatable and early tool for assessing supraspinatus tendon degeneration in asymptomatic patients with T2DM. Active control of the condition of diabetes patients and regular upper limb exercise might help delay the degeneration of supraspinatus tendons.

**Supplementary Information:**

The online version contains supplementary material available at 10.1186/s12891-025-08864-w.

## Introduction

The incidence of degenerative rotator cuff disease increases with age [1], and the supraspinatus tendon is the most commonly impaired due to its special anatomical location, which has become the focus of clinical attention [2]. The incidence of shoulder disease in type II diabetes mellitus (T2DM) patients is approximately five times greater than that in healthy people [3]. Xu K [4] used a T2DM rat model to demonstrate that the histopathology of the rotator cuff showed disorganized or even broken collagen fibrils and a disorganized extracellular matrix. Therefore, there is a consensus that diabetes increases the possibility of tendon injury [5–7]. Therefore, for timely clinical intervention or rehabilitation, early detection of rotator cuff degeneration in T2DM patients is important for preventing rotator cuff tears and reducing long-term functional impairment [8, 9].

However, when early rotator cuff degeneration occurs, patients often have no evident shoulder symptoms, making detection via physical examination difficult. Magnetic resonance imaging [10, 11] can reveal tendon degeneration and fatty infiltration, but it is relatively poor at showing calcification and bony structures and easily produces artefacts. Moreover, Shingh [12] used conventional ultrasound and reported that supraspinatus tendons in T2DM patients exhibit thickening, a poorly displayed fibrous texture, and irregular edges, but inflammation and aging might also lead to these signs. Thus, a more accurate and sensitive method to evaluate early rotator cuff degeneration in diabetic patients is preferred.

Degenerative tendons exhibit a decrease in mechanical properties when subjected to various applied loads [13], which can be assessed using ultrasound elastography [14]. A meta-analysis [15] showed a strong correlation (R2 = 0.785) between the ultrasound elasticity measurement and the ultimate tendon load. Shear wave elastography (SWE) is a type of ultrasound elastography that yields a quantitative shear wave velocity (SWV) [14]. The SWV is greater in harder tissues and vice versa. The SWV correlates well with the measurements of mechanical instruments in the porcine infraspinatus [16], rabbit gastrocnemius [17], and human cadaveric patellar tendons [18]. SWE has been used to identify the extent of supraspinatus tendon tears [19], observe the elastic variation of rotator cuff after corticosteroid injections [20] and assess Achilles tendon stiffness in T2DM patients [21].

Previously, our research team demonstrated that ultrasound elastography is useful for evaluating supraspinatus tendon stiffness [22, 23]. However, no study has assessed the changes in the mechanical properties of the supraspinatus tendon in diabetic patients before an actual tendon tear. In this study, early degeneration of the supraspinatus tendon was defined as the absence of clinical symptoms such as shoulder pain but the presence of imaging evidence of supraspinatus tendon abnormalities (ultrasonic signs or quantitative indicators). This multicenter study used conventional ultrasound and SWE to compare the ultrasonography, thickness, blood flow grading, and SWV of the supraspinatus tendon between T2DM patients without shoulder pain and healthy subjects to evaluate the feasibility and repeatability of SWE for the assessment of early degeneration of the supraspinatus tendon in patients with T2DM. The key variables influencing supraspinatus tendon thickness and the SWV were also explored by regression analysis.

## Materials and Methods

This multicenter study (ClinicalTrials.gov, March 15th 2023, ID: NCT05769712) was conducted from March 2021 to October 2022 and received approval from the institutional review board of the hospitals on November 5th 2020; all subjects signed the informed consent form. Seven hospitals in China participated in this multicenter study.

### Study subjects and operators

Subjects were consecutively recruited, including healthy people and diabetic patients who met the 2020 ADA clinical diagnosis guidelines, and all of them underwent assessment of shoulder function to confirm their asymptomatic status. The inclusion and exclusion criteria are shown in Table 1.

**Table 1:** Inclusion and exclusion criteria

Inclusion criteria	Healthy people	①Age > 18 years; ②Non-professional athletes; ③No history of shoulder and neck pain; ④Ultrasonic examination (-)
	Diabetic patients	①Age > 18 years; ②Non-professional athletes; ③Patients who met the clinical diagnosis and treatment guidelines for diabetes and were diagnosed with diabetes (2020 ADA guidelines: In the presence of typical diabetic symptoms and random plasma glucose ≥ 11.1 mmol/L, fasting plasma glucose ≥ 7.0 mmol/L, or patients without typical diabetic symptoms with 2 h postprandial plasma glucose ≥ 11.1 mmol/L); ④ Medical history ≥ 2 years
Exclusion criteria	All subjects	①Pregnant women or postpartum within 1 year; ②Shoulder pain of active or passive cervical spine movement; ③History of upper extremity trauma; ④History of shoulder surgery or treatment, intra-articular injection, use of glucocorticoids, estrogens, quinolones and cholesterol drugs; ⑤Shoulder joint fear test (when shoulder joint abduction was 90°and external rotation was slowly increased, the subjects'fear expression was positive); ⑥Evidence of adhesive shoulder bursitis, such as significant limitation of passive range of motion in two shoulder planes; ⑦Systemic autoimmune disease, metabolic disease, endocrine disease except diabetes, psoriasis; ⑧End stage renal disease; ⑨Unable to complete the relevant movements and positions

Seven radiologists with more than two years of experience in musculoskeletal ultrasound conducted the examinations at the seven hospitals. They were specially trained for the operation of this project and passed the operation assessment of the whole process.

### Equipment and parameter setting

Seven centers used the same study protocol and ultrasound instrument (Aplio i800; Canon Medical Systems i18LX5 linear probe). Examination parameters were uniformly set to SHOULDER-SWE: differential harmonics: 18 MHz; dynamic range: 65; image display depth: 3–4 cm; focus placed at the level of the supraspinatus tendon; and gain adjusted to the appropriate size (initial gain: 79).

- Two-dimensional (2D) ultrasound conditions: frame rate: 32 fps; spatial composite imaging A: 2; precise imaging P: 4.
- Advanced Dramatic Flow (ADF) imaging conditions: frame rate: 11 fps; frequency DF: 11; color gain DF: 40; filter: 5; and scale setting: 2.1 cm/s.
- The SWE conditions were as follows: frame rate: 0.4 fps; SW frequency: 5 MHz; shear wave filter (SF): 3; sampling frame should include the supraspinatus tendon, deltoid and humeral tuberosity; and diameter of the region of interest (ROI): 1 mm.

### Ultrasound examination methods

#### Body position and standard measuring section

**Fig. 1:**
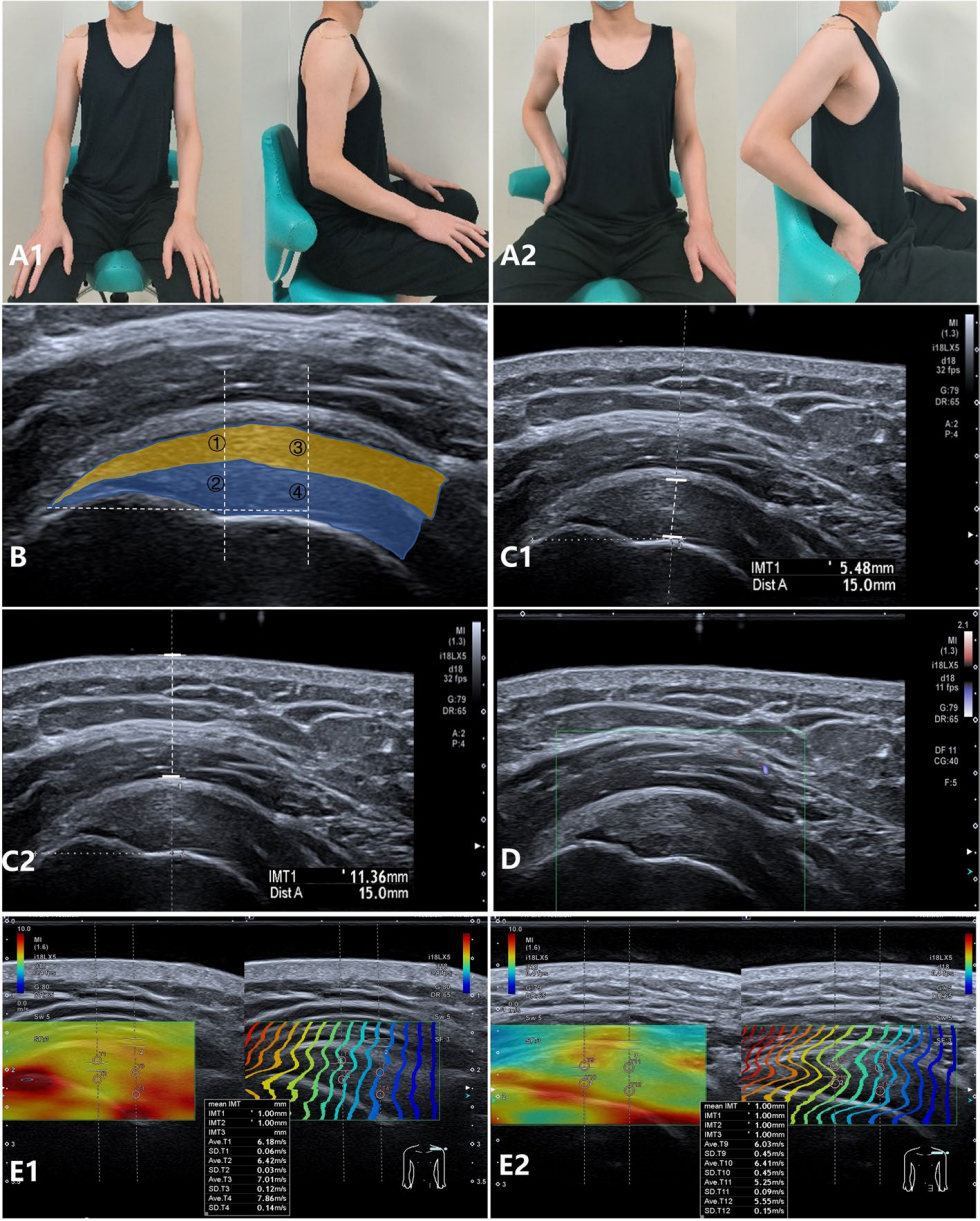
Schematic diagram of the body position and ultrasound evaluation of the supraspinatus tendon. Note: (**A**) Subjects'body position: (A1) frontal and lateral view of relaxed position #1, with the palms of both hands placed naturally on the thighs; (A2) frontal and lateral view of tension position #2, with the palms of the right hand placed on the lateral edge of the ipsilateral anterior superior iliac spine, keeping fingers together and pointing forward. **B** Schematic diagram illustrating the SWV measurement of the supraspinatus tendon. The tendon was stratified based on its depth from the skin surface: a superficial part was indicated by the yellow layer, and a deep part by the blue layer. The distal end was demarcated at 10 mm from the humeral tuberosity attachment site, whereas the proximal end was set at 15 mm. The sequence of measurements was as follows: ①upper distal, ②deep distal, ③upper proximal, and ④deep proximal. **C** Schematic diagram of the 2D ultrasound measurements for the supraspinatus tendon and the soft tissue layer situated superior to it: (C1) depicts the supraspinatus tendon thickness (indicated by the dotted line), measuring 5.48 mm, and (C2) illustrates the thickness of the soft tissue above the supraspinatus tendon (also indicated by the dotted line), which is 11.36 mm. **D** Schematic diagram of the internal tendon's blood flow signal assessment using advanced dramatic flow imaging, indicating a grade 0 blood flow. **E** The SWE mode setting defines red as relatively hard tissue and blue as relatively soft tissue. For example, the supraspinatus tendon region of a 66-year-old healthy female subject (E1) is shown in yellow (suggesting a high elasticity value), and the region of a 63-year-old diabetic female subject (E2) is shown in light green (suggesting a low stiffness value). The mean SWVs of these healthy and diabetic subjects were 6.86 and 5.81 m/s, respectively

In order to minimize the interference of motion on measurement, subjects were instructed to avoid upper extremity weight-bearing activities for one hour before the examination. The examination position was divided into two types: (1) relaxed position #1 (Fig. 1.A1), with the palms of both hands placed naturally on the thighs; and (2) tension position #2 (Fig. 1.A2), with the palms placed on the lateral edge of the ipsilateral anterior superior iliac spine, keeping fingers together and pointing forward. The shoulder joint was left in the neutral position for adduction and abduction.

Bilateral shoulder joint ultrasound examination: The probe was placed parallel to the long axis of the supraspinatus tendon, with the acoustic beam perpendicular to the tendon as much as possible to exclude anisotropic artifacts. A unified standard section was defined when the"beak-like"structure at the end of the supraspinatus tendon was clear and the acromion disappeared. In this section, 2D ultrasound, ADF, and SWE images of the supraspinatus tendon were acquired. In the supraspinatus tendon image (Fig. 1.B), the distal and proximal ends were defined at 10 and 15 mm from the humeral tuberosity attachment point, respectively. The supraspinatus tendons were evenly divided into upper (shallow) and lower (deep) layers. During the entire examination, an ultrasound gel pad was added to the supraspinatus tendon in the shoulder.

#### 2D ultrasound

At the proximal end, the thickness of the supraspinatus tendon was measured at a tangent line perpendicular to the humeral surface (Fig. 1.C1). The soft tissue thickness was measured between the top surface of the tendon and the epidermis perpendicular to the horizontal line (Fig. 1.C2) three times each. The shoulder joint was also evaluated for changes in the following ultrasound signs: supraspinatus tendon uneven thickening, uneven echogenicity, unclear fibrous texture of the tendon, intratendon calcification foci, tendon rim irregularity, bony cortical irregularity, and subacromial-deltoid bursa uneven thickening.

#### Assessment of tendon blood flow

The blood flow within the supraspinatus tendon was assessed according to the modified Adler grade [24] (Fig. 1.D): grade 0: no blood flow signal; grade I: little blood flow signal (1 to 2 dots or bars); grade II: moderate blood flow signal (3 to 4 dots or 1 bar); and grade III: abundant blood flow signal (> 4 bars).

#### SWE detection of the tendon

The supraspinatus tendon was placed in the center of the elastic sampling frame, keeping the probe stable and avoiding applying pressure. After activation in the SWE mode, elastic images were generated in the double-frame mode (Fig. 1.E). After the image quality was qualified (uniform, equidistant, and smooth color curve), the SWVs of the upper and lower layers perpendicular to the horizontal line were measured at the distal and proximal ends, respectively. The ROI was placed in the middle of the upper or deep layers of the tendon, avoiding the bone surface and bursa. Patients with localized abnormalities such as localized hypoechoicity or calcification within the tendon were excluded. The supraspinatus tendon SWV was evaluated three times. Each interval should be longer than two minutes. The order of measurements was upper distal, lower distal, upper proximal, and lower proximal (Fig. 1.B).

### Statistical analysis

SPSS 25.0 was used to analyse the data for normality, and χ^2^ tests were used. Quantitative data are expressed as the means ± standard deviations (SD), and categorical data are expressed as numbers and percentages (N, %). The probability of positive ultrasound signs in the supraspinatus tendon was compared between diabetic and healthy subjects using χ^2^ tests. Intragroup correlation coefficients (ICCs) were used to evaluate the intra- and intergroup consistency of supraspinatus tendon thickness and the SWV. Based on the 95% confidence intervals (95% CIs) of the ICCs, values less than 0.5, between 0.5 and 0.75, between 0.75 and 0.9, and greater than 0.90 indicate poor, moderate, good, and excellent reliability, respectively [25]. The average value was taken for statistical analysis if the difference between the three repeated measurements was not statistically significant.

To overcome the selection bias between groups, SPSS propensity score matching was used to perform 1:1 matching between healthy people and diabetic patients with a calliper value of 0.03. The difference in the bilateral supraspinatus tendon SWV at different positions was evaluated by paired sample t tests, and the difference in the supraspinatus tendon SWV at different measurement sites was evaluated by the Friedman test. Single-factor and multifactor linear regression analysis models were used to evaluate the factors influencing the thickness and SWV of the supraspinatus tendon before and after matching. The multivariate regression model included factors with *p* < 0.05 in the univariate linear regression analysis. *p* < 0.05 was considered to indicate statistical significance.

According to our previous study [22], the standard deviation of the supraspinatus tendon SWV was 0.82 cm/s. With a two-sided test with α = 0.05 and a tolerance error of 0.10 cm/s, a sample size of n = 259 was calculated using PASS15; assuming a missing visit rate of 10% and sample loss after matching of 10%, at least 320 subjects needed to be collected.

## Results

### Subject characteristics

**Fig. 2:**
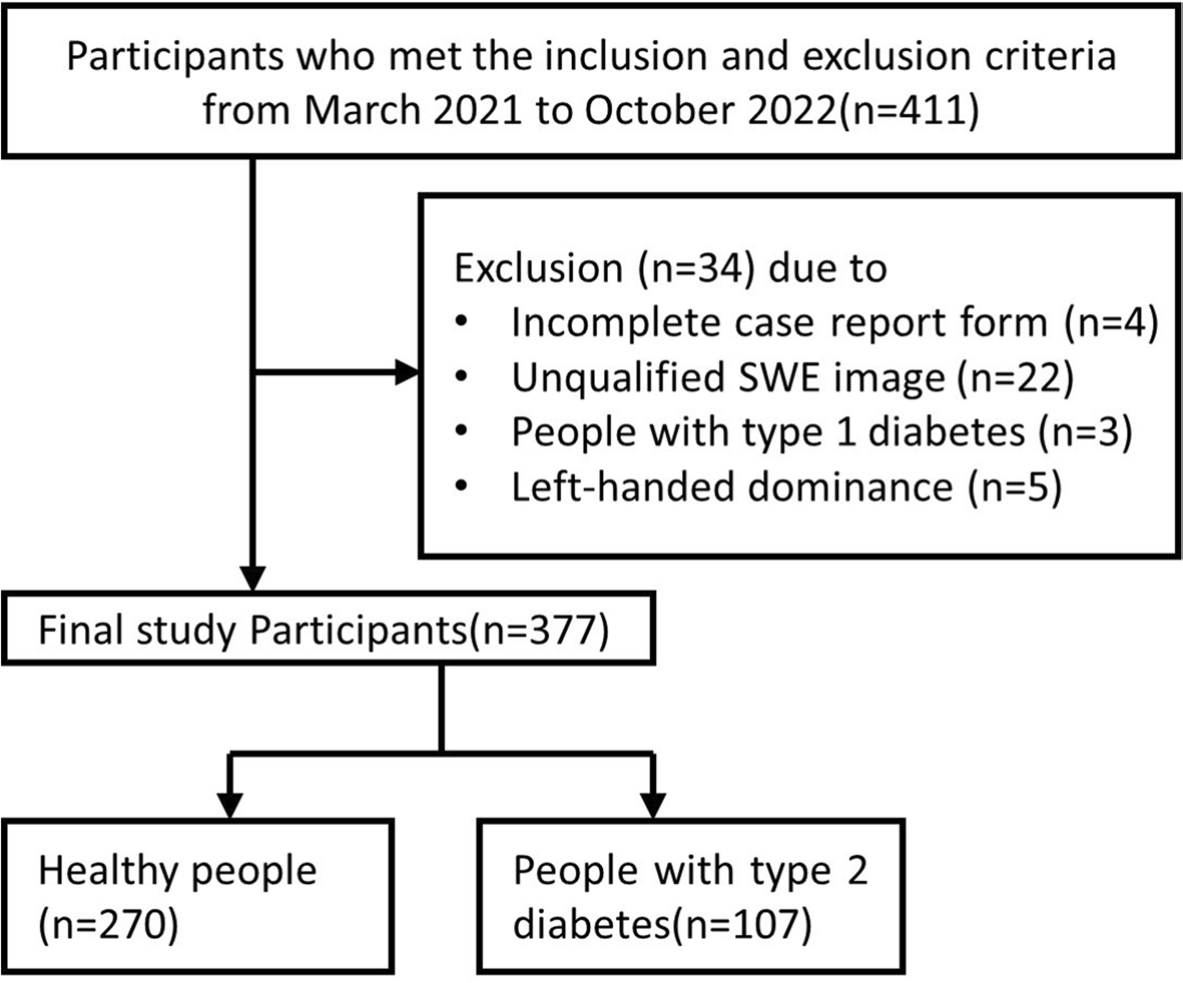
Recruitment flowchart of the participants

A total of 411 subjects were first enrolled, after excluding some cases (Fig. 2), 377 subjects, including 107 T2DM patients (mean age 53.2 ± 12.2 years, 56 men) and 270 healthy people (mean age 37.6 ± 13.3 years, 106 men), were ultimately included. 2TDM was the only type of diabetes specified in this paper.

Age, gender, ethnicity, BMI, and long-term smoking status significantly differed between the two groups (all *p* < 0.05) before propensity score matching. After propensity score matching (90 pairs matched), there was no significant difference in any of the variables between the two groups (all *p* > 0.05), and an intergroup balance was achieved. The baseline characteristics of the subjects are shown in Table 2.

**Table 2:** Subject demographic variables and clinical characteristics

Characteristics	Before matching			After matching		
	**Normal** (*n* = 270)	**Diabetics** (*n* = 107)	***p* value**	**Normal** (*n* = 90)	**Diabetics** (*n* = 90)	***p* value**
Matched variable						
Age (years old)			< 0.001			0.450
18–50	214(79.3)	40(37.4)		35(38.9)	40(44.4)	
> 50	56(20.7)	67(62.6)		55(61.1)	50(55.6)	
Gender			0.021			0.765
Male	106(39.3)	56(52.3)		49(54.4)	47(52.2)	
Female	164(60.7)	51(47.7)		41(45.6)	43(47.8)	
Race			0.009			0.136
Han	171(63.3)	52(48.6)		50(55.6)	40(44.4)	
Non-Han	99(36.7)	55(51.4)		40(44.4)	50(55.6)	
BMI (kg/m^2^)			< 0.001			0.408
Normal (18.5 ~ 25.0)	235(87.0)	75(70.1)		67(74.4)	62(68.9)	
Overweight (> 25.0)	35(13.0)	32(29.9)		23(25.6)	28(31.1)	
Whether smoking			0.002			0.816
No	257(95.2)	92(86.0)		80(88.9)	79(87.8)	
Yes (> 10 years)	13(4.8)	15(14.0)		10(11.1)	11(12.2)	
Whether exercise			0.616			0.999
No	240(88.9)	97(90.7)		79(87.8)	79(87.8)	
Yes (> 5 h/week)	30(11.1)	10(9.3)		11(12.2)	11(12.2)	
Unmatched variables						
Soft tissue thickness (mm)						
Non-dominant side	9.6 ± 2.0	10.4 ± 2.1	0.797	9.8 ± 1.9	10.4 ± 2.1	0.089
Dominant side	9.9 ± 2.2	10.9 ± 2.4	0.148	10.2 ± 2.4	10.8 ± 2.5	0.167
Diabetes duration (years)	—^*^	7.1 ± 5.1		—^*^	6.5 ± 4.6	
FPG (mmol/L)	—^*^	10.9 ± 4.7		—^*^	11.2 ± 4.7	
HbA1C (mmol/L)	—^*^	9.5 ± 2.7		—^*^	9.6 ± 2.6	
Whether use insulin						
No	—^*^	69(64.5)		—^*^	60(66.7)	
Yes (> 2 years)	—^*^	38(35.5)		—^*^	30(33.3)	

Note: Data for continuous variables are shown as (X ± s). Dichotomous variables are shown as the number of subjects, and the data in parentheses are percentages for that column. The P value represents the difference between T2DM patients and healthy subjects. BMI, body mass index; FPG, fasting plasma glucose; HbA1c, haemoglobin A1c. —* indicates that this indicator was not collected from healthy subjects

### Sonographic comparison of thickness and blood flow in T2DM patients and healthy subjects

The results of routine ultrasonography showed that 59.8% of T2DM patients and 82.2% of healthy subjects had normal shoulder joint findings. The probability of ultrasonic performance associated with degenerative disease was significantly greater in 2TDM patients than in healthy subjects (Table 3). T2DM patients were more likely than healthy subjects to have poorly displayed fibre texture in the supraspinatus tendon (16.8 vs. 6.5%, *p* < 0.001), foci of calcification within the tendon (6.1 vs. 1.9%, *p* = 0.005), and uneven thickening of the bursa (8.9 vs. 2.6%, *p* < 0.001). However, the probability of uneven supraspinatus tendon thickening (4.7 vs. 1.9%, *p* = 0.055) and internal blood flow signals (3.7 vs. 1.7%, *p* = 0.060) did not differ between the two groups.

**Table 3:** Comparison of positive ultrasound signs between T2DM and healthy subjects

Ultrasonic signs	Normal (*n* = 540)	Diabetic (*n* = 214)	*P* value
Unevenly thickened tendon	10/540(1.9%)	10/214 (4.7%)	0.055
uneven echo	35/540(6.5%)	26/214 (12.1%)	0.015
unclear fibrous texture	35/540(6.5%)	26/214 (16.8%)	< 0.001
calcification within the tendon	10/540(1.9%)	13/214(6.1%)	0.005
Irregular tendon margins	1/540(0.2%)	12/214(5.6%)	< 0.001
Unsmooth cortex of bone	9/540(1.7%)	17/214(7.9%)	< 0.001
unevenly thickened subacromial—subdeltoid bursa	14/540(2.6%)	19/214(8.9%)	< 0.001
blood flow signals within the tendon	9/540(1.7%)	8/214 (3.7%)	0.060

Note: Number of supraspinatus tendons with positive ultrasound signs/total number of tendons in this group, with proportions in parentheses. The *p* value represents the difference between T2DM and healthy subjects

### The consistency of the SWE tendon assessment

Intragroup consistency: The ICCs were used to evaluate the consistency between three repeated measurements of supraspinatus tendon thickness and the SWV (Table S1). The results showed that the ICCs of thickness were > 0.90 (*p* < 0.001), indicating excellent reliability. Moreover, 87.5% of ICCs of SWV were between 0.75 and 0.9 (*p* < 0.001), indicating good reliability. The other 50% ranged from 0.50 to 0.75 (*p* < 0.001), showing moderate reliability. The difference between the three repeated measurements was not statistically significant, and using the average was considered valid.

Intergroup consistency: After matching the population, age, gender, ethnicity, and BMI data for each hospital, balanced baseline distribution data were obtained, and the upper distal SWV of the supraspinatus tendon at relaxation position #1 was selected for assessment. Six samples were randomly selected from each of the seven hospitals. The results showed good consistency (ICC = 0.757, *p* < 0.001) for assessing the upper distal SWV in the supraspinatus tendon from each hospital.

### Reference values for supraspinatus tendon thickness and the SWV

The reference values are shown in Table S2a-j. The thickness and SWV of the bilateral supraspinatus tendons of the subjects were evaluated in different groups and at different positions. Considering all variables consistent, the tendons were thicker, and the SWV was lower on the dominant side than on the nondominant side (all *p* < 0.05), as well as in position #1 than in position #2 (all *p* < 0.05).

Furthermore, to obtain a meaningful and stable result, the supraspinatus tendon SWV at position 1 on the dominant side (which is easily available on all shoulder joints) was chosen because it is a natural relaxation position without the influence of different abduction angles and acromion coverage and because of the high usage of the dominant side. The differences in SWV among the measurement sites did not differ (all *p* > 0.05) (Table S3).

### Comparison of thickness between T2DM patients and healthy subjects

With diabetes diagnosis as the independent variable, multifactor linear regression analysis was performed on the supraspinatus tendon thickness of all subjects (position #1, dominant hand). The results before and after matching are shown in Table 4.

**Table 4:** Regression analysis of factors influencing supraspinatus tendon thickness

**Characteristics**	**Before matching**			**After matching**
	**β (95% CI)**	***P* value**	**β (95% CI)**	***P* value**
Group				
Normal	1(reference)		1(reference)	
Diabetics	0.143(−0.086,0.372)	0.219	0.240(−0.036,0.516)	0.088
Age (years old)				
18–50	1(reference)		1(reference)	
> 50	0.320(0.105,0.534)	**0.004**	0.346(0.059,0.633)	**0.018**
Gender				
Male	1(reference)		1(reference)	
Female	−0.367(−0.565,−0.170)	** < 0.001**	−0.204(−0.505,0.097)	0.182
Race				
Han	1(reference)		1(reference)	
Non-Han	0.816(0.619,1.012)	** < 0.001**	0.669(0.376,0.962)	** < 0.001**
BMI (kg/m^2^)				
Normal (18.5 ~ 25.0)	1(reference)		1(reference)	
Overweight (> 25.0)	0.669(0.422,0.916)	** < 0.001**	0.591(0.284,0.897)	** < 0.001**
Whether smoking				
No	1(reference)		1(reference)	
Yes (> 10 years)	−0.014(−0.391,0.364)	0.943	0.163(−0.300,0.625)	0.488
Whether exercise				
No	1(reference)		1(reference)	
Yes (> 5 h/week)	−0.308(−0.613,−0.004)	0.052	−0.479(−0.913,0.046)	0.060

Note: BMI, body mass index

Before and after matching, no significant correlation was detected between diabetes and supraspinatus tendon thickness (*p* > 0.05). The thickness of the supraspinatus tendon in subjects > 50 years old and overweight/obese (BMI ≥ 25.0) subjects was greater than that in the corresponding control group (*p* < 0.05).

Before matching, the results showed that females were thinner than males (*p* < 0.001) but did not differ after matching (*p* = 0.182). No significant correlation was detected between supraspinatus tendon thickness, long-term smoking status, or regular upper limb exercise (*p* > 0.05).

### Comparison of the SWV between T2DM patients and healthy subjects

Due to the lack of blood supply at the"distal"end of the supraspinatus tendon [26] and the significantly greater incidence of lower layer tears than upper layer tears [27], the lower distal SWV at position 1 and on the dominant side was selected for analysis. Before and after matching (Table 5), the supraspinatus tendon SWV was lower in T2DM patients than in healthy subjects (*p* ≤ 0.001) (example shown in Fig. 1.E). The box scatter plots of comparing the lower distal SWV between the two matched groups are shown in Figure S4.

**Table 5:** Regression analysis of factors influencing the supraspinatus tendon SWV

**Characteristics**	**Before matching**			**After matching**
	**β (95% CI)**	***P* value**	**β (95% CI)**	***P* value**
Group				
Normal	1(reference)		1(reference)	
Diabetics	−0.541(−0.799,−0.282)	** < 0.001**	−0.529(−0.827,−0.232)	**0.001**
Age (years old)				
18–50	1(reference)		1(reference)	
> 50	0.028(−0.214,0.270)	0.820	0.086(−0.222,0.394)	0.581
Gender				
Male	1(reference)		1(reference)	
Female	−0.208(−0.431,0.015)	**0.068**	−0.327(−0.649,−0.005)	**0.047**
Race				
Han	1(reference)		1(reference)	
Non-Han	−0.535(−0.756,−0.314)	** < 0.001**	−0.603(−0.916,−0.289)	** < 0.001**
BMI (kg/m^2^)				
Normal (18.5 ~ 25.0)	1(reference)		1(reference)	
Overweight (> 25.0)	0.295(−0.014,0.604)	0.061	0.278(−0.105,0.660)	0.153
Whether smoking				
No	1(reference)		1(reference)	
Yes (> 10 years)	−0.002(−0.427,0.423)	0.993	−0.161(−0.657,0.334)	0.522
Whether exercise				
No	1(reference)		1(reference)	
Yes (> 5 h/week)	0.360(0.016,0.703)	**0.040**	0.314(−0.150,0.778)	**0.048**
Soft tissue thickness (mm)	−0.024(−0.081,0.034)	0.423	−0.019(−0.101,0.063)	0.647

Note: BMI, body mass index

In addition, the SWV was lower in Chinese non-Han population (mainly Chinese Zhuang population) than in Chinese Han population (*p* < 0.001). The SWV in subjects with regular upper limb exercise > 5 h/week was greater than that in subjects without upper limb exercise habits (*p* < 0.05). Nevertheless, the SWV was not significantly correlated with age, BMI, long-term smoking status, or soft tissue thickness (*p* > 0.05). Additionally, before matching, there was no significant difference in the SWV between the genders. However, the female SWV was lower (*p* = 0.047) than the male SWV after matching.

Subgroup analysis was performed on 90 diabetic patients after matching, and the variables with *p* < 0.05 mentioned above were incorporated into the regression model. The model also included the disease course, fasting blood glucose, haemoglobin A1c, and whether insulin was used for more than two years. Patients with a long course of diabetes had a lower supraspinatus tendon SWV (*p* = 0.004). Moreover, the SWV was lower in patients who used insulin to control blood glucose for more than two years (*p* = 0.045). However, fasting blood glucose (*p* = 0.282) and haemoglobin A1c (*p* = 0.877) were not significantly correlated with the SWV (Table S5).

## Discussion

This study assessed the application of SWE on the supraspinatus tendon in nonshoulder pain T2DM patients. T2DM was associated with a significantly lower tendon SWV, and the intra- and intergroup consistency was good for SWE assessment. This finding contributes to discovering the early effects of diabetes on the tendon, which can lay the foundation for clinical risk prediction and timely intervention.

One study [19] demonstrated that supraspinatus tendon tears affect tendon elasticity with a reduced SWV. To avoid the effects of tendon tears or inflammation, patients with abnormal shoulder function assessments and ultrasound indications of rotator cuff tears were excluded from the present study. Thus, SWE can be used to more objectively and accurately assess nonshoulder pain symptoms associated with purely degenerative mechanical properties of the supraspinatus tendon in T2DM patients. The results showed an increased degenerative probability and reduced SWV of supraspinatus tendons in T2DM patients compared to healthy subjects. Sneha et al. [21] also found softer Achilles tendons in diabetic patients using SWE. These results suggested that the supraspinatus tendons of T2DM patients exhibit softening earlier than those of healthy people, and the incidence of rotator cuff tears will continue to be tracked. The reason may lie in the abnormal proliferation, apoptosis and remodeling ability of tendon cells in the presence of chronic hyper-glycemia and advanced glycosylation end products, thus damaging the homeostasis and healing of tendon [28, 29]. The mechanisms might involve persistent diabetes promoting AGE and RAGE upregulation [30], causing increased NOX and ROS production [31] and contributing to the accumulation of glycosylated products in rotator cuff tissue [32], eventually leading to tendon fibrosis and local inflammation. In addition, high glucose also altered tendon homeostasis by down-regulating of the AMPK/Egr1 pathway and the expression of downstream tendon-related genes in tenocytes [33].

Herein, the supraspinatus tendon SWV was not correlated with FPG or HbA1c in T2DM patients, while patients with longer course of disease and insulin use had a significantly lower SWV, indicating lower tendon stiffness. FPG and HbA1c reflect short-term blood glucose levels, with immediate values and three months of control [34]. The diabetic patients included in this study were all well controlled by long-term medication, but had poor control recently. Therefore, the abnormal blood glucose level with large fluctuations in the recent period could not fully reflect the daily blood glucose situation of the patient in the long-term course of disease. In contrast, the use of diabetes course and insulin use time to measure the long-term chronic effects of diabetes on the body is more stable and objective. Therefore, the decrease in the supraspinatus tendon SWV in T2DM patients might be associated with long-term disease effects. As some studies have shown, diabetic tendinopathy is not an overuse injury of normal tendons, but the end result of long-term metabolic degradation [35]. Patients with insulin use and diabetes duration (average 5.3 years) had an increased risk of tendinopathy [36]. And poor glycemic control after rotator cuff tear repair is associated with an increased risk of re-tear [37, 38]. These finding suggested that proactively controlling the disease is conducive to reducing complications and might help slow the rate of degeneration and decrease the mechanical properties of supraspinatus tendons.

The guidelines for musculoskeletal ultrasound developed by the European Society of Musculoskeletal Radiology [39] state that routine ultrasound can screen for shoulder disorders. Shingh [12] et al. suggested that the supraspinatus tendon was thicker in diabetic patients than in healthy people, but we did not find a difference between the two groups, possibly because 80% of Shingh's diabetic patients were male. However, we confirmed that male supraspinatus tendons were thicker than female supraspinatus tendons before matching. Thus, gender imbalance might interfere with thickness assessment. In the ADF mode, 96.3% of the T2DM patients had a grade 0 blood flow signal within the supraspinatus tendon, which was not significantly greater than that in healthy people. This might be because supraspinatus tendons are blood-deprived structures, and persistently high blood glucose concentrations might prevent the production of vascular endothelial growth factor [40] and thicken the capillary basement membrane [41], resulting in less intratendon blood flow.

Furthermore, upper limb exercise might affect the mechanical properties of the supraspinatus tendon. Pre-matching, post-matching, and subgroup analysis indicated that the supraspinatus tendon SWV was greater in the regular upper limb exercise group (> 5 h/week) than in the group without exercise habits, suggesting greater tendon stiffness. Mechanically, exercise might promote the synthesis of growth factors and tendon collagen and decrease the glycosylation response, increasing tendon stiffness [42, 43]. These results might suggest that regular upper limb exercise can help delay the progression of supraspinatus tendon degeneration, both diabetic and healthy individuals. Therefore, early intervention is preferred when the SWV of the tendon is below the normal reference range. Previous studies have suggested that diabetes-related tendinopathy is managed in a manner similar to overuse tendinopathy. Early physical therapy referrals to specific exercise programs are essential, a comprehensive exercise prescription should be designed including strength, flexibility and aerobic fitness. If tendinitis is present, NSAID or corticosteroid injections are needed [35, 44]. This also reminds that personal life habits should also be considered during individualized assessments.

Our research group [22] previously used the virtual touch tissue imaging quantification (VTIQ) technique, a shear wave ultrasound imaging technique, to quantitatively evaluate the supraspinatus tendon in healthy subjects. That study showed that the SWV values of the deep tendon layers were found to be higher than those of the superficial layers, and the SWV values at the distal end of the tendon were greater than those at the proximal end. However, the supraspinatus tendon SWV did not differ at different measurement points in the present study. This might be because the addition of the ultrasound gel pad in this study reduced the effect of probe pressurization on tissue stiffness, leading to more stable propagation of shear waves within the tissue and improved measurement accuracy. On the other hand, the VTIQ technique uniformly shows values exceeding 10 m/s as"out of detection range'', making it difficult to display the quantitative value accurately. In contrast, the SWE technique used in this study can provide specific values above this threshold, which makes the results more objective.

Previous studies have shown [19, 45] that SWE has good feasibility for assessing the stiffness of tendons. This multicenter study has good representativeness with its large sample size covered on a geographically diverse basis, reaffirming the good reproducibility and stability of SWE in assessing the consistency of three repeated measurements across operators for the same type of subjects. At present, there is a scarcity of studies exploring the application of SWE for the supraspinatus tendon in predicting early tendinopathy. This research may fill this gap by offering preliminary insights into risk prediction and early intervention strategies for tendinopathy, thereby establishing a foundation for future investigative work.

However, this study also has limitations. First, the small sample size of individuals who were left-handed and had type 1 diabetes could not reflect the effect of different dominant hands and types of diabetes. Second, since the supraspinatus tendon changes in T2DM patients were not confirmed by biopsy or surgery, the SWV cut-off value cannot be provided to indicate early degeneration, but we provide reference values for SWV in different populations. In addition, the study did not collect information on subjects' occupational activities, familial musculoskeletal conditions, or hormones, limiting the diversity of results to some extent. Finally, this study found significant differences in tendon’s SWV between Chinese Han and non-Han population, and further exploration of ethnic differences in SWV is needed in the future.

## Conclusion

SWE provides an accurate and early assessment of supraspinatus tendon degeneration in T2DM patients without shoulder pain. Compared with those of healthy people, supraspinatus tendons are softened in T2DM patients even without shoulder pain symptoms. Active control of the condition of diabetes patients and regular upper limb exercise might help delay the degeneration of supraspinatus tendons. This study provides a new method and perspective for monitoring diabetic tendinopathy and lays a foundation for protecting supraspinatus tendon quality in T2DM patients and for making early clinical intervention decisions.

## Supplementary Information

Supplementary Material 1. Table S1: Consistency analysis of various operators in repeated measurements of supraspinatus tendon thickness and SWV

Supplementary Material 2. Table S2a: The reference values of bilateral supraspinatus tendon thickness at different body positions in normal subjects. Note: Data for continuous variables are shown as ($\overline{\text{x} }$±s). Table S2b: The reference values of bilateral supraspinatus tendon thickness at different body positions in diabetic subjects. Note: Data for continuous variables are shown as ($\overline{\text{x} }$±s). Table S2c: The reference values of the bilateral supraspinatus tendons’ upper distal SWV at different body positions in normal subjects. Note: Data for continuous variables are shown as ($\overline{\text{x} }$±s). Table S2d: The reference values of the bilateral supraspinatus tendons’ upper distal SWV at different body positions in diabetic subjects. Note: Data for continuous variables are shown as ($\overline{\text{x} }$±s). Table S2e: The reference values of the bilateral supraspinatus tendons’ lower distal SWV at different body positions in normal subjects. Note: Data for continuous variables are shown as ($\overline{\text{x} }$±s). Table S2f The reference values of the bilateral supraspinatus tendons’ lower distal SWV at different body positions in diabetic subjects. Note: Data for continuous variables are shown as ($\overline{\text{x} }$±s). Table S2g The reference values of the bilateral supraspinatus tendons’ upper proximal SWV at different body positions in normal subjects. Note: Data for continuous variables are shown as ($\overline{\text{x} }$±s). Table S2h The reference values of the bilateral supraspinatus tendons’ upper proximal SWV at different body positions in diabetic subjects. Note: Data for continuous variables are shown as ($\overline{\text{x} }$±s). Table S2i The reference values of the bilateral supraspinatus tendons’ lower proximal SWV at different body positions in normal subjects. Note: Data for continuous variables are shown as ($\overline{\text{x} }$±s). Table S2j The reference values of the bilateral supraspinatus tendons’ lower proximal SWV at different body positions in diabetic subjects. Note: Data for continuous variables are shown as ($\overline{\text{x} }$±s).

Supplementary Material 3. Figure S4: Box scatter plots of the bilateral supraspinatus tendon’ lower distal SWV at different body positions. Note: (A) Box scattered plot of supraspinatus tendon’s lower distal SWV in dominant side, position #1. Under this condition, the median and Inter-quartile range of diabetic patients’ SWV were 4.78 and 1.37m/s, respectively, and those of normal people were 5.06 and 1.46m/s, respectively. (B) Box scattered plot of supraspinatus tendon’s lower distal SWV in dominant side, position #2. Under this condition, the median and Inter-quartile range of diabetic patients’ SWV were 6.29 and 1.56m/s, respectively, and those of normal people were 6.63 and 1.57m/s, respectively. (C) Box scattered plot of supraspinatus tendon’s lower distal SWV in non-dominant side, position #1. Under this condition, the median and Inter-quartile range of diabetic patients’ SWV were 5.00 and 1.66m/s, respectively, and those of normal people were 5.57 and 1.51m/s, respectively. (D) Box scattered plot of supraspinatus tendon’s lower distal SWV in non-dominant side, position #2. Under this condition, the median and Inter-quartile range of diabetic patients’ SWV were 6.61 and 1.85m/s, respectively, and those of normal people were 6.74 and 1.99m/s, respectively.

Supplementary Material 4. Table S5: Regression analysis of factors influencing the supraspinatus tendon SWV in type 2 diabetic patients.

Supplementary Material 5. Table S3: Comparison of the SWV between different measurement sites of the supraspinatus tendon on position #1 on the dominant side Note: Data for continuous variables are shown as (

$$\overline{\text{x} }$$±s).

## Data Availability

No datasets were generated or analysed during the current study.
