# Supplementary material for: Shear wave elastography of the supraspinatus tendon with early degeneration in asymptomatic type II diabetes mellitus patients: a multicenter study
Source: BMC Musculoskelet Disord. 2025 Jul 4;26:637. doi: 10.1186/s12891-025-08864-w (PMC12232052; doi:10.1186/s12891-025-08864-w)
Supplement: Supplementary file 1 — Supplementary Material 1. Table S1: Consistency analysis of various operators in repeated measurements of supraspinatus tendon thickness and SWV [file 12891_2025_8864_MOESM1_ESM.docx]

**Table** **S1 Consistency analysis of various operators in repeated measurements of supraspinatus tendon thickness and SWV**

| **Target variable** | **time** | | | $\overline{\text{x}}$**±s** | **ICC** |
| --- | --- | --- | --- | --- | --- |
| Non-dominant side, position#1 thickness (mm) | | 1 | 5.38 ± 1.00 | | 0.983 |
|  |  | 2 | 5.37 ± 1.02 | |  |
|  |  | 3 | 5.37 ± 1.04 | |  |
| Non-dominant side, position#2 thickness (mm) | | 1 | 4.42 ± 0.78 | | 0.977 |
|  |  | 2 | 4.41 ± 0.77 | |  |
|  |  | 3 | 4.41 ± 0.77 | |  |
| Dominant side, position#1  thickness (mm) | | 1 | 5.64 ± 1.08 | | 0.990 |
|  |  | 2 | 5.64 ± 1.09 | |  |
|  |  | 3 | 5.62 ± 1.09 | |  |
| Dominant side, position#2  thickness (mm) | | 1 | 4.53 ± 0.80 | | 0.985 |
|  |  | 2 | 4.54 ± 0.81 | |  |
|  |  | 3 | 4.54 ± 0.80 | |  |
| Non-dominant side, position#1 upper distal SWV(m/s) | | 1 | 5.11 ± 1.18 | | 0.849 |
|  |  | 2 | 5.05 ± 1.15 | |  |
|  |  | 3 | 5.04 ± 1.17 | |  |
| Non-dominant side, position#1 lower distal SWV(m/s) | | 1 | 5.21 ± 1.26 | | 0.802 |
|  |  | 2 | 5.22 ± 1.15 | |  |
|  |  | 3 | 5.22 ± 1.22 | |  |
| Non-dominant side, position#1  upper proximal SWV(m/s) | | 1 | 5.03 ± 1.22 | | 0.845 |
|  |  | 2 | 5.00 ± 1.23 | |  |
|  |  | 3 | 5.04 ± 1.31 | |  |
| Non-dominant side, position#1  lower proximal SWV(m/s) | | 1 | 5.20 ± 1.29 | | 0.848 |
|  |  | 2 | 5.24 ±1.28 | |  |
|  |  | 3 | 5.26 ± 1.32 | |  |
| Non-dominant side, position#2 upper distal SWV(m/s) | | 1 | 6.51 ± 1.32 | | 0.767 |
|  |  | 2 | 6.41 ± 1.30 | |  |
|  |  | 3 | 4.45 ± 1.32 | |  |
| Non-dominant side, position#2 lower distal SWV(m/s) | | 1 | 6.54 ± 1.31 | | 0.772 |
|  |  | 2 | 6.53 ± 1.28 | |  |
|  |  | 3 | 6.51 ± 1.34 | |  |
| Non-dominant side, position#2  upper proximal SWV(m/s) | | 1 | 6.55 ± 1.38 | | 0.772 |
|  |  | 2 | 6.65 ± 1.38 | |  |
|  |  | 3 | 6.59 ± 1.37 | |  |
| Non-dominant side, position#2  lower proximal SWV(m/s) | | 1 | 6.78 ± 1.48 | | 0.780 |
|  |  | 2 | 6.79 ± 1.50 | |  |
|  |  | 3 | 6.90 ± 1.54 | |  |
| Dominant side, position#1 upper distal SWV(m/s) | | 1 | 5.03 ± 1.11 | | 0.800 |
|  |  | 2 | 5.04 ± 1.09 | |  |
|  |  | 3 | 5.09 ± 1.14 | |  |
| Dominant side, position#1 lower distal SWV(m/s) | | 1 | 4.94 ± 1.18 | | 0.837 |
|  |  | 2 | 4.90 ± 1.19 | |  |
|  |  | 3 | 4.94 ± 1.22 | |  |
| Dominant side, position#1  upper proximal SWV(m/s) | | 1 | 5.00 ± 1.20 | | 0.801 |
|  |  | 2 | 4.97 ± 1.20 | |  |
|  |  | 3 | 4.97 ± 1.21 | |  |
| Dominant side, position#1  lower proximal SWV(m/s) | | 1 | 4.85 ± 1.31 | | 0.848 |
|  |  | 2 | 4.83 ± 1.33 | |  |
|  |  | 3 | 4.88 ± 1.32 | |  |
| Dominant side, position#2 upper distal SWV(m/s) | | 1 | 6.35 ± 1.19 | | 0.727 |
|  |  | 2 | 6.35 ± 1.18 | |  |
|  |  | 3 | 6.32 ± 1.22 | |  |
| Dominant side, position#2 lower distal SWV(m/s) | | 1 | 6.30 ± 1.28 | | 0.767 |
|  |  | 2 | 6.29 ± 1.25 | |  |
|  |  | 3 | 6.27 ± 1.34 | |  |
| Dominant side, position#2  upper proximal SWV(m/s) | | 1 | 6.29 ± 1.26 | | 0.747 |
|  |  | 2 | 6.33 ± 1.30 | |  |
|  |  | 3 | 6.38 ± 1.40 | |  |
| Dominant side, position#1  lower proximal SWV(m/s) | | 1 | 6.38 ± 1.38 | | 0.761 |
|  |  | 2 | 6.43 ± 1.37 | |  |
|  |  | 3 | 6.40 ± 1.41 | |  |

Note: SWV, shear wave velocity.
