# Supplementary material for: Shear wave elastography of the supraspinatus tendon with early degeneration in asymptomatic type II diabetes mellitus patients: a multicenter study
Source: BMC Musculoskelet Disord. 2025 Jul 4;26:637. doi: 10.1186/s12891-025-08864-w (PMC12232052; doi:10.1186/s12891-025-08864-w)
Supplement: Supplementary file 2 — Supplementary Material 2. Table S2a: The reference values of bilateral supraspinatus tendon thickness at different body positions in normal subjects. Note: Data for continuous variables are shown as ($\overline{\text{x} }$x¯±s). Table S2b: The reference values of bilateral supraspinatus tendon thickness at different body positions in diabetic subjects. Note: Data for continuous variables are shown as ($\overline{\text{x} }$x¯±s). Table S2c: The reference values of the bilateral supraspinatus tendons’ upper distal SWV at different body positions in normal subjects. Note: Data for continuous variables are shown as ($\overline{\text{x} }$x¯±s). Table S2d: The reference values of the bilateral supraspinatus tendons’ upper distal SWV at different body positions in diabetic subjects. Note: Data for continuous variables are shown as ($\overline{\text{x} }$x¯±s). Table S2e: The reference values of the bilateral supraspinatus tendons’ lower distal SWV at different body positions in normal subjects. Note: Data for cont [file 12891_2025_8864_MOESM2_ESM.zip › Table S2a.docx]

**Table S2a The reference values of bilateral supraspinatus tendon thickness at different body positions** **in normal subjects**

| **Thickness**  (mm) | **Normal**（n=90） | | | | |
| --- | --- | --- | --- | --- | --- |
|  | Position#1 | Position#2 | *P* | Effect size | 95%CI |
| Non-dominant side | 5.46±0.96 | 4.59±0.79 | <0.001 | 0.990 | (0.744,1.027) |
| Dominant side | 5.78±1.06 | 4.70±0.82 | <0.001 | 1.140 | (0.892,1.178) |
| *P* | <0.001 | 0.049 |  | | |
| Effect size | 0.316 | 0.137 |  |  |  |
| 95%CI | (-0.433 -0.188) | (-0.261, -0.060) |  |  |  |

Note: Data for continuous variables are shown as ($\overline{\text{x}}$±s).
