# Supplementary material for: Shear wave elastography of the supraspinatus tendon with early degeneration in asymptomatic type II diabetes mellitus patients: a multicenter study
Source: BMC Musculoskelet Disord. 2025 Jul 4;26:637. doi: 10.1186/s12891-025-08864-w (PMC12232052; doi:10.1186/s12891-025-08864-w)
Supplement: Supplementary file 2 — Supplementary Material 2. Table S2a: The reference values of bilateral supraspinatus tendon thickness at different body positions in normal subjects. Note: Data for continuous variables are shown as ($\overline{\text{x} }$x¯±s). Table S2b: The reference values of bilateral supraspinatus tendon thickness at different body positions in diabetic subjects. Note: Data for continuous variables are shown as ($\overline{\text{x} }$x¯±s). Table S2c: The reference values of the bilateral supraspinatus tendons’ upper distal SWV at different body positions in normal subjects. Note: Data for continuous variables are shown as ($\overline{\text{x} }$x¯±s). Table S2d: The reference values of the bilateral supraspinatus tendons’ upper distal SWV at different body positions in diabetic subjects. Note: Data for continuous variables are shown as ($\overline{\text{x} }$x¯±s). Table S2e: The reference values of the bilateral supraspinatus tendons’ lower distal SWV at different body positions in normal subjects. Note: Data for cont [file 12891_2025_8864_MOESM2_ESM.zip › Table S2b.docx]

**Table S2b The reference values of bilateral supraspinatus tendon thickness at different body positions in diabetic subjects**

| **Thickness**  **(mm)** | **Diabetics**（n=90） | | | | |
| --- | --- | --- | --- | --- | --- |
|  | Position#1 | Position#2 | *P* | Effect size | 95%CI |
| Non-dominant side | 5.89±0.90 | 4.68±0.73 | <0.001 | 1.477 | (1.034,1.290) |
| Dominant side | 6.09±1.02 | 4.80±0.77 | <0.001 | 1.427 | (-0.790,1.190) |
| *P* | 0.007 | 0.024 |  | | |
| Effect size | 0.208 | 0.160 |  |  |  |
| 95%CI | (-0.304, -0.047) | (-0.128,1.853) |  |  |  |

Note: Data for continuous variables are shown as ($\overline{\text{x}}$±s).
