# Supplementary material for: Shear wave elastography of the supraspinatus tendon with early degeneration in asymptomatic type II diabetes mellitus patients: a multicenter study
Source: BMC Musculoskelet Disord. 2025 Jul 4;26:637. doi: 10.1186/s12891-025-08864-w (PMC12232052; doi:10.1186/s12891-025-08864-w)
Supplement: Supplementary file 2 — Supplementary Material 2. Table S2a: The reference values of bilateral supraspinatus tendon thickness at different body positions in normal subjects. Note: Data for continuous variables are shown as ($\overline{\text{x} }$x¯±s). Table S2b: The reference values of bilateral supraspinatus tendon thickness at different body positions in diabetic subjects. Note: Data for continuous variables are shown as ($\overline{\text{x} }$x¯±s). Table S2c: The reference values of the bilateral supraspinatus tendons’ upper distal SWV at different body positions in normal subjects. Note: Data for continuous variables are shown as ($\overline{\text{x} }$x¯±s). Table S2d: The reference values of the bilateral supraspinatus tendons’ upper distal SWV at different body positions in diabetic subjects. Note: Data for continuous variables are shown as ($\overline{\text{x} }$x¯±s). Table S2e: The reference values of the bilateral supraspinatus tendons’ lower distal SWV at different body positions in normal subjects. Note: Data for cont [file 12891_2025_8864_MOESM2_ESM.zip › Table S2h.docx]

| **upper proximal**  **SWV** (m/s) | **Diabetics**（n=90） | | | | | |
| --- | --- | --- | --- | --- | --- | --- |
|  | Position#1 | Position#2 | | *P* | Effect size | 95%CI |
| Non-dominant side | 5.08±1.21 | 6.72±1.44 | <0.001 | | 1.233 | (-2.069, -1.507) |
| Dominant side | 4.91±1.21 | 6.40±1.34 | <0.001 | | 1.167 | (-1.606, -1.121) |
| *P* | <0.001 | 0.005 |  | | | |
| Effect size | 0.140 | 0.230 |  |  |  |  |
| 95%CI | (-0.413,0.049) | (-0.064,0.549) |  |  |  |  |

**Table S2h The reference values of the bilateral supraspinatus tendons’ upper proximal SWV at different body positions in diabetic subjects**

Note: Data for continuous variables are shown as ($\overline{\text{x}}$±s).
