# Supplementary material for: Shear wave elastography of the supraspinatus tendon with early degeneration in asymptomatic type II diabetes mellitus patients: a multicenter study
Source: BMC Musculoskelet Disord. 2025 Jul 4;26:637. doi: 10.1186/s12891-025-08864-w (PMC12232052; doi:10.1186/s12891-025-08864-w)
Supplement: Supplementary file 2 — Supplementary Material 2. Table S2a: The reference values of bilateral supraspinatus tendon thickness at different body positions in normal subjects. Note: Data for continuous variables are shown as ($\overline{\text{x} }$x¯±s). Table S2b: The reference values of bilateral supraspinatus tendon thickness at different body positions in diabetic subjects. Note: Data for continuous variables are shown as ($\overline{\text{x} }$x¯±s). Table S2c: The reference values of the bilateral supraspinatus tendons’ upper distal SWV at different body positions in normal subjects. Note: Data for continuous variables are shown as ($\overline{\text{x} }$x¯±s). Table S2d: The reference values of the bilateral supraspinatus tendons’ upper distal SWV at different body positions in diabetic subjects. Note: Data for continuous variables are shown as ($\overline{\text{x} }$x¯±s). Table S2e: The reference values of the bilateral supraspinatus tendons’ lower distal SWV at different body positions in normal subjects. Note: Data for cont [file 12891_2025_8864_MOESM2_ESM.zip › Table S2j.docx]

**Table S2j The reference values of the bilateral supraspinatus tendons’ lower proximal SWV at different body positions in diabetic subjects**

| **lower proximal**  **SWV** (m/s) | **Diabetics**（n=90） | | | | | |
| --- | --- | --- | --- | --- | --- | --- |
|  | Position#1 | Position#2 | | *P* | Effect size | 95%CI |
| Non-dominant side | 5.14±1.21 | 6.98±1.53 | <0.001 | | 1.334 | (-2.123, -1.545) |
| Dominant side | 4.97±1.30 | 6.51±1.33 | <0.001 | | 1.171 | (-1.851, -1.328) |
| *P* | <0.001 | <0.001 |  | | | |
| Effect size | 0.135 | 0.328 |  |  |  |  |
| 95%CI | (-0.046,0.422) | (0.140,0.727) |  |  |  |  |

Note: Data for continuous variables are shown as ($\overline{\text{x}}$±s).
