# Supplementary figures and images for: Shear wave elastography of the supraspinatus tendon with early degeneration in asymptomatic type II diabetes mellitus patients: a multicenter study
Source: BMC Musculoskelet Disord. 2025 Jul 4;26:637. doi: 10.1186/s12891-025-08864-w (PMC12232052; doi:10.1186/s12891-025-08864-w)

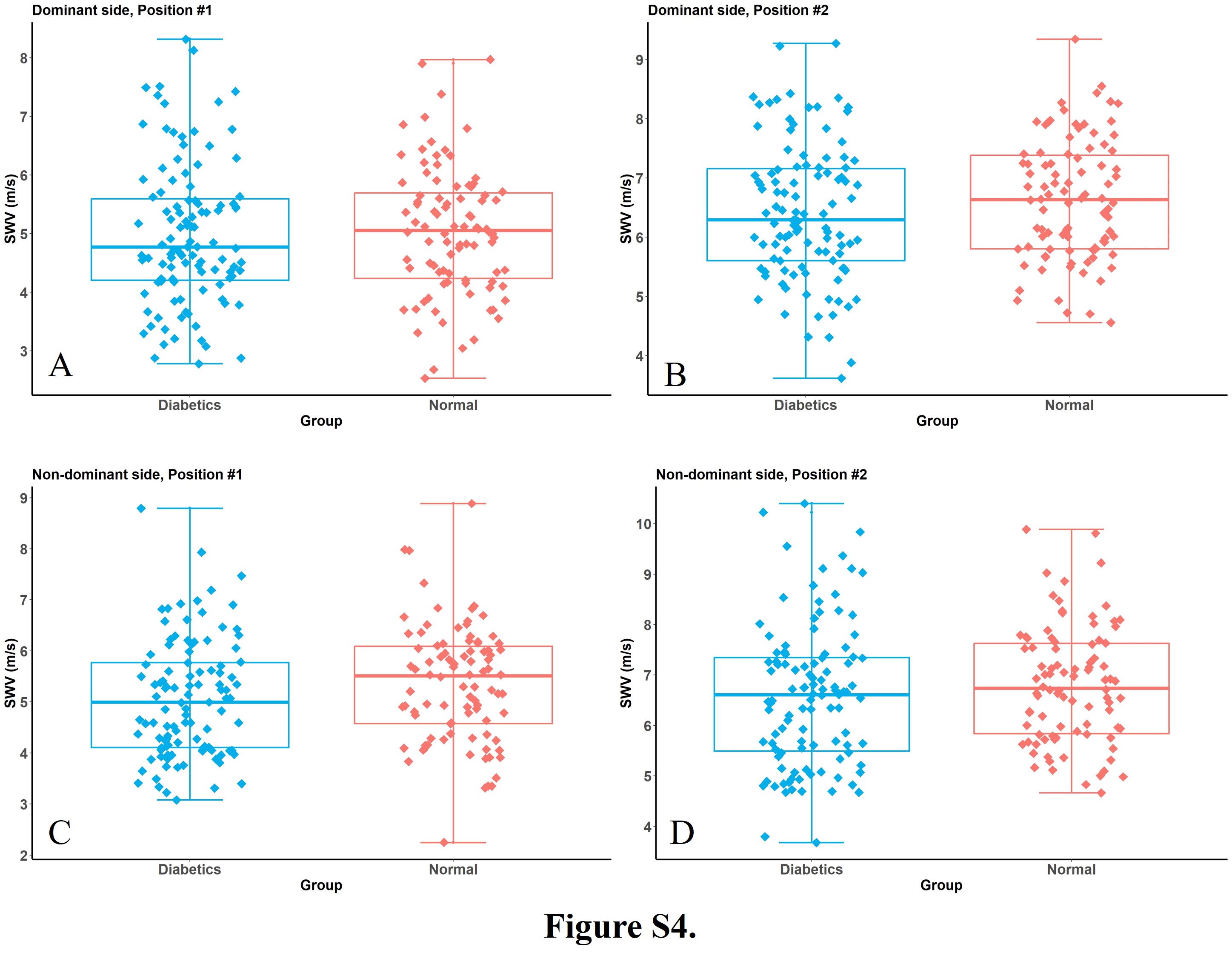

Supplement: Supplementary file 3 — Supplementary Material 3. Figure S4: Box scatter plots of the bilateral supraspinatus tendon’ lower distal SWV at different body positions. Note: (A) Box scattered plot of supraspinatus tendon’s lower distal SWV in dominant side, position #1. Under this condition, the median and Inter-quartile range of diabetic patients’ SWV were 4.78 and 1.37m/s, respectively, and those of normal people were 5.06 and 1.46m/s, respectively. (B) Box scattered plot of supraspinatus tendon’s lower distal SWV in dominant side, position #2. Under this condition, the median and Inter-quartile range of diabetic patients’ SWV were 6.29 and 1.56m/s, respectively, and those of normal people were 6.63 and 1.57m/s, respectively. (C) Box scattered plot of supraspinatus tendon’s lower distal SWV in non-dominant side, position #1. Under this condition, the median and Inter-quartile range of diabetic patients’ SWV were 5.00 and 1.66m/s, respectively, and those of normal people were 5.57 and 1.51m/s, respectively. (D) Box scattered plot of supraspinatus tendon’s lower distal SWV in non-dominant side, position #2. Under this condition, the median and Inter-quartile range of diabetic patients’ SWV were 6.61 and 1.85m/s, respectively, and those of normal people were 6.74 and 1.99m/s, respectively. [file 12891_2025_8864_MOESM3_ESM.jpg]
