# Supplementary material for: Shear wave elastography of the supraspinatus tendon with early degeneration in asymptomatic type II diabetes mellitus patients: a multicenter study
Source: BMC Musculoskelet Disord. 2025 Jul 4;26:637. doi: 10.1186/s12891-025-08864-w (PMC12232052; doi:10.1186/s12891-025-08864-w)
Supplement: Supplementary file 4 — Supplementary Material 4. Table S5: Regression analysis of factors influencing the supraspinatus tendon SWV in type 2 diabetic patients. [file 12891_2025_8864_MOESM4_ESM.docx]

**Table S5** **Regression analysis of factors influencing the supraspinatus tendon SWV in type 2 diabetic patients**

| **Characteristics** | **β (95% CI)** | | ***P* value** |
| --- | --- | --- | --- |
| **Gender** |  | |  |
| Male | 1(reference) | |  |
| Female | -0.263(-0.743,0.217) | | 0.279 |
| **Race** |  | |  |
| Han | 1(reference) | |  |
| Non-Han | -0.764(-1.339,-0.190) | | **0.010** |
| **Whether exercise** |  | |  |
| No | 1(reference) | |  |
| Yes (> 5 h/week) | 0.934(0.149,1.719) | | **0.020** |
| **Diabetes duration** (years) | -0.080(-0.133,-0.027) | | **0.004** |
| **FPG** (mmol/L) | -0.032 (-0.090,0.027) | | 0.282 |
| **HbA1C** (mmol/L) | 0.008 (-0.089,0.104) | | 0.877 |
| **Whether use insulin** | |  |  |
| No | 1(reference) | |  |
| Yes (> 2 years) | -0.546(-1.080,0.012) | | **0.045** |

Note: SWV, shear wave velocity; BMI, body mass index; FPG, fasting plasma glucose; HbA1c, haemoglobin A1c
