# Supplementary material for: Shear wave elastography of the supraspinatus tendon with early degeneration in asymptomatic type II diabetes mellitus patients: a multicenter study
Source: BMC Musculoskelet Disord. 2025 Jul 4;26:637. doi: 10.1186/s12891-025-08864-w (PMC12232052; doi:10.1186/s12891-025-08864-w)
Supplement: Supplementary file 5 — Supplementary Material 5. Table S3: Comparison of the SWV between different measurement sites of the supraspinatus tendon on position #1 on the dominant side Note: Data for continuous variables are shown as ( [file 12891_2025_8864_MOESM5_ESM.docx]

**Table S3 Comparison of the SWV between different measurement sites of the supraspinatus tendon on position #1 on the dominant side**

| **measurement site** | **Normal**（n=90） | **Diabetics**（n=90） |
| --- | --- | --- |
| upper distal | 5.14±0.95 | 5.14±1.10 |
| lower distal | 5.04±1.09 | 4.95±1.20 |
| upper proximal | 5.14±1.08 | 5.08±1.21 |
| lower proximal | 5.03±1.30 | 4.97±1.30 |
| *P* | 0.100 | 0.141 |
| Effect size | 0.024 | 0.044 |

Note: Data for continuous variables are shown as ($\overline{\text{x}}$±s).
